# 605 Revision Surgery Following Severe Frostbite Compared to Similar Hand and Foot Burns

**DOI:** 10.1093/jbcr/irac012.233

**Published:** 2022-03-23

**Authors:** Alexandra Coward, Rachel M Nygaard, Frederick W Endorf

**Affiliations:** Hennepin County Medical Center, Minneapolis, Minnesota; Hennepin County Medical Center, Minneapolis, Minnesota; Hennepin Healthcare, Minneapolis, Minnesota

## Abstract

**Introduction:**

Severe frostbite is associated with high levels of morbidity through loss of digits or limbs. The current practice is to salvage as much of the limb/digit as possible with the use of thrombolytic and adjuvant therapies. Sequalae from amputation can include severe nerve pain and poor wound healing requiring revision surgery. The aim of this study was to examine the rate of revision surgery after primary amputation and compare this to revision surgery in isolated hand/foot burns.

**Methods:**

Frostbite and burn patients from 2006 to 2019 were identified in the prospectively maintained database at a single urban burn and trauma center. Patients with primary amputations related to isolated hand/foot burns or frostbite were included in the study. Descriptive statistics included Student’s T-test and Fisher’s Exact test.

**Results:**

A total of 63 patients, 54 frostbite injuries and 9 isolated hand or foot burns, met inclusion criteria for the study. The rate of revision surgery was similar following frostbite and burn injury (24% vs 33%, P=0.681). There were no significant differences in age, gender, or LOS on the primary hospitalization. Neither the impacted limb nor the presence of infection or cellulitis on primary amputation were associated with future need for revision surgery. Of the 16 patients requiring revision surgery, 5 (31%) required additional debridement alone, 6 (38%) required re-amputation alone, and 5 required both. A total of 6 patients (38%) had cellulitis or infection at the time of revision surgery. Time from primary surgery to revision ranged from 4 days to 3 years.

**Conclusions:**

Planned, delayed primary amputation is a mainstay of frostbite management. To our knowledge, this is the first assessment of revision surgery in the setting of severe frostbite injury. Our observed rate of revision surgery following frostbite injury did not differ significantly from revision surgery in the setting of isolated hand or foot burns. This study brings up important questions of timing and surgical planning in these complex patients that will require a multicenter collaborative study.

